# Cow's Milk and Rickets

**Published:** 1908-06-06

**Authors:** 


### EDITOR'S LETTER-BOX.

### COW'S MILK AND RICKETS.

SIR,—Three and three make six. As Dr. Stephens opened the discussion, matters will be even if I close it. I notice that he has shifted his standpoint a good deal, but I fear he cannot even be left his assumption that milk from pregnant cattle is inferior in quality. What happens in a state of nature? If the herd is to produce young yearly, must not necessarily a good deal of suckling be done by pregnant animals? Why does the cow have a natural *oestrus* a few weeks after delivery? Perhaps the dairyman, although he takes care to "dry off" the cow some time before delivery, exploits her a little too much; but there seems nothing unnatural in milking her when pregnant. In conclusion, let me say that I am not a fervent admirer of the "Harley Street Pantheon," to use Dr. Stephens's rather happy phrase.

Yours, etc.,

London, May 30. J.

